# Lower Serum Androstenedione Levels in Pre-Rheumatoid Arthritis versus Normal Control Women: Correlations with Lower Serum Cortisol Levels

**DOI:** 10.1155/2013/593493

**Published:** 2013-05-22

**Authors:** Alfonse T. Masi, Kevin B. Elmore, Azeem A. Rehman, Robert T. Chatterton, Ned J. Goertzen, Jean C. Aldag

**Affiliations:** ^1^Department of Medicine, University of Illinois College of Medicine (UICOMP), One Illini Drive, Peoria, IL 61656, USA; ^2^University of Illinois College of Medicine at Peoria, Peoria, IL 61656, USA; ^3^Northwestern University (NWU), Feinberg School of Medicine, Chicago, IL 60611, USA

## Abstract

Serum adrenal androgens (AAs), including androstenedione (Δ4A) and dehydroepiandrosterone sulfate (DHEAS), have been reported to be lower in female rheumatoid arthritis (RA) patients with early disease. Few data are available on hormonal status of women before the onset of clinical rheumatoid arthritis (pre-RA). A broad baseline panel of serum adrenal and sex steroids was compared in 36 female pre-RA to 144 matched cohort control (CN) subjects to determine differences in their mean values and in patterns of hormonal correlations. Study subjects having lower versus higher baseline serum cortisol levels than the total group's mean value were also analyzed separately to investigate differences in their hormonal levels and correlational patterns. In total subjects, mean (±SE) Δ4A level (nmol/L) was lower (*P* = 0.018) in 28 pre-RA cases (6.4 ± 0.40) versus 108 CN (7.8 ± 0.28). The significant (*P* = 0.013) difference was restricted to 9 pre-RA versus 53 CN subjects having lower cortisol levels (5.6 ± 0.73 versus 8.0 ± 0.42 nmol/L, resp.). In total subjects, no significant difference was found between study subjects in their bivariate correlations of the hormonal panel variables, unlike results found in the subgroups stratified by lower versus higher cortisol levels. A subgroup of pre-RA females may have relative adrenal cortical insufficiency, as reflected by lower Δ4A, especially observed among those subjects with lower cortisol levels.

## 1. Introduction

Relative insufficiency of adrenal glucocorticoid (GC) and androgenic-anabolic (AA) hormones has been suspected to increase the risk of developing rheumatoid arthritis (RA) and to contribute to its multifactorial neuroendocrine immune (NEI) pathogenesis [1–8]. The characteristic age- and sex-specific incidence patterns of RA support a possible constitutional deficiency of adrenal cortical or sex hormones in a subset of susceptible women. The female-to-male (F : M) risk ratio of persons developing RA is approximately 2 : 1 during the juvenile and older ages but is significantly increased to about 5 : 1 during the female reproductive years [9]. Of further note, risk of RA onset increases with age in adults, particularly among females. The preceding risk data imply that males have relative protection over females during all ages, but particularly in the younger and middle adult years [9]. A recent study indicated that early age at menopause (≤45 years) was associated with the subsequent risk of developing RA [10]. The hazard remained significant after adjusting for smoking, educational level, and length of breastfeeding [10]. Available data imply that the woman's risk of developing RA may be affected by relative insufficiency of both adrenal cortical GC and AA steroids as well as by accelerated aging of their ovarian hormonal status [1–10].

 A quantitative study of adrenal steroid urinary metabolites from 8 early disease premenopausal RA women not treated with GC drugs and 8 healthy matched controls under varied baseline, physiological adrenal stimulation, and metyrapone GC-inhibiting conditions supported AA deficiency in the patients [11]. Significant differences were found only in the 11-deoxy-17-ketosteroid AA steroids: dehydroepiandrosterone (DHEA), androsterone, and etiocholanolone [11]. Subsequent studies of serum DHEA and its sulfate (DHEAS) in early disease premenopausal onset women found lower mean levels of these AA biomarkers than in matched controls [5, 8, 12, 13]. A nested case-control cohort study showed that definitely low baseline serum DHEAS levels (<0.68 *μ*mol/L), assayed in independent reference laboratories, were present a mean of 11 years before premenopausal onset in 3 (30%) of 10 pre-RA versus 1 (2.7%) of 37 matched CN subjects (*P* = 0.026) [14]. Recently, the same cohort study [15] revealed that definitely low baseline serum cortisol levels had occurred in a greater minority of pre-RA than CN females (11% versus 1.4%, resp., *P* = 0.016). In addition, none of 28 pre-RA women had serum androstenedione (Δ4A) levels in the upper normal range (≥7.5 nmol/L) versus 26 (24%) of 108 CN women (*P* = 0.010). The lower levels of urinary AA metabolites, serum DHEA, and DHEAS in premenopausal early RA patients and the recent findings of baseline lower serum cortisol, as well as a truncated upper range of Δ4A levels in a minority of pre-RA versus CN female cohort subjects, have prompted this further analysis of a comprehensive panel of adrenal cortical and sex steroids in our pre-RA and CN cohort subjects [15, 16].

## 2. Materials and Methods

### 2.1. The RA Precursors Study (RAPS) Database at This Institution

The RA Precursors Study (RAPS) was initiated at this institution in late 1991, following donation of baseline personal data and serum samples from the pre-RA cases and matched CN cohort subjects by “Operation CLUE I,” a community-wide prospective study [19–21]. The 1974 CLUE I base cohort had enrolled 12,381 females of Washington County, Maryland, USA. The RAPS female database currently includes 180 study subjects, 36 Caucasian pre-symptomatic RA (pre-RA) cases at their 1974 baseline entry, and 144 matched cohort CN subjects, in a ratio of 1 pre-RA: 4 CN. The UICOMP Institutional Review Board has approved this research for assurance of confidentiality. 

The pre-RA cases in this study conform to The European League Against Rheumatism (EULAR) recommendations [22]. All baseline pre-RA cases were diagnosed and confirmed in the practice of the sole rheumatologist in the cohort community, who used the American College of Rheumatology (ACR) 1987 revised classification criteria [23]. Following the 1974 cohort entry, clinical onsets of RA in our study females occurred within 3 to 18 years (1977 to 1992), after a median of 11 years. None of the matched comparison subjects had a diagnosis of RA in the community rheumatologist's practice. The non-RA subjects (CN) were matched to the pre-RA cases on race (all Caucasians) and usually within one year of age at entry. Also, the CN subjects were the closest in chronological sequence of enrollment in the cohort, analogous to another case-control study [24]. 

In 1992, after clinical onset and diagnosis of the first set of baseline pre-RA females, 4 cohort CN subjects were matched to each female case on entry age and sex to permit more specific search for hormonal determinants of RA, other than those already known demographic risk factors [9, 25]. Case or control subjects who had known cancer diagnoses during follow-up were excluded from the RAPS database. Their sera were reserved to study cancer biomarkers, which is the primary purpose of Operation CLUE [19–21].

### 2.2. The Hormonal Reference Laboratory Performed Assays, in 1992 and 1994 (Table 1)

The mean concentrations of the original reported assays performed by the referral laboratory in the first 1992 and second 1994 sets of subjects are indicated in Table 1, as well as other statistical values. The baseline-stored (−70°C) cohort sera were always analyzed in matched sets of 1 pre-RA and 4 CN, without knowledge of subject status. As funding was secured during the interval of 1992 to 1994, the first (1992) and second (1994) sets of study subjects' sera were sequentially donated by project CLUE for the hormonal assays in the referral laboratory. The first set of study subjects were 14 baseline pre-RA and 56 CN females. Their frozen sera were sent by CLUE to the Immunoassay Core Facility at Northwestern University (NWU) for hormonal assays in 1992 [16]. One subject had no sera available, but the other 69 had mostly sufficient sera to assay the full panel of hormones; a limited number lacked DHEA (*n* = 6), Δ4A (*n* = 5), and testosterone (*n* = 9) assays (Table 1). Serum estrone levels were only assayed in the first set females. The second set of female cohort subjects were 22 pre-RA and 88 CN subjects, whose frozen sera were sent from CLUE I to the NWU laboratory, in 1994. Those sera were sufficient to perform 4 hormonal tests completely (corticosterone, deoxycortisol, cortisol, and DHEAS) or almost completely (progesterone) (Table 2). Also, they were shared with other laboratories which performed immunological assays [16]. Two-thirds of the other tests were completed, but one-half of estradiol assays, which had the lowest concentrations, requiring greater amount of sera.

### 2.3. Specific Assays Were Developed for a Comprehensive Panel of Serum Steroids (Figure 1)

**Figure 1:**
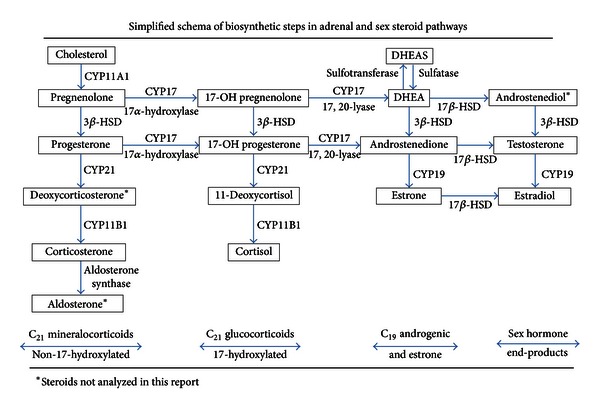
A simplified schema of the major adrenal and sex steroids, emphasizing the biosynthetic pathways leading to mineralocorticoids, glucocorticoids, adrenal androgens (AAs), and sex steroids. Initial conversion from cholesterol to pregnenolone is acutely controlled by ACTH, which chronically activates genes promoting other enzymes in the biosynthetic steps. The adrenal cortical volume increases during pre-pubertal adrenarche until young adulthood, particularly in the zona reticularis (ZR), along with its greater AA production, including DHEA, DHEAS, and androstenedione. Individual variability occurs in serum DHEAS levels, being lower in adult females than males, particularly after menopause (adrenopause). Serum AAs progressively decline with aging and are presumably accompanied by diminishing ZR mass. To the contrary, cortisol is derived from the zona fasciculata (ZF) and its levels remain stable over the ages, as does the ZF mass.

A comprehensive panel of adrenal and sex steroids were assayed (Figure 1) using a procedure developed specifically for this study to permit measurements of 12 steroids in duplicate from 1 mL of serum. The fractionation procedure permitted assay of each steroid in greater sensitivity than would otherwise be detectable without fractionation. The method also provided an additional purification step, adding to the specificity of the procedure. 

Serum (1.0 mL) was diluted by addition of 1.0 mL of 4 M urea. ^3^H-tracers (5,000 cpm) of representative steroids, estrone (E1), testosterone (T), and progesterone (prog), were added and the solution was heated for 30 min at 60°C to denature the protein. Steroids were extracted by the use of an ODS cartridge (SepPak, Millipore, Inc., Billerica, MA). The cartridge was washed with 3 mL methanol followed by 6 mL of distilled water. The serum was aspirated through the cartridge. Retention of the steroids was complete, as negligible loss of the ^3^H-tracers into the effluent had occurred. A 7 mL of water wash was discarded, and the conjugated steroids (androstanediol glucuronide and DHEAS) were eluted with 10 mL of 47% aqueous methanol (Fraction A1). The second fraction was eluted with 12 mL of 60% aqueous methanol (Fraction B1). This fraction contained cortisol (F), deoxycortisol (DF), corticosterone (cmpd B), deoxycorticosterone (DOC), estrone (E1), and estradiol (E2). The third fraction was eluted with 12 mL of methanol: water: acetonitrile (55 : 35 : 10). It contained androstenedione (Δ4A), dehydroepiandrosterone (DHEA), 17-hydroxyprogesterone (17-OHprog), 17-hydroxypregnenolone (17-OHpreg), and testosterone (T) (Fraction C1). The fourth fraction was eluted with 12 mL of methanol and it contained progesterone (prog) and pregnenolone (preg) (Fraction D1). 

The aqueous component of each of the first two fractions was removed by adding an equal volume of water and transferring to a second ODS cartridge. The flow-through was discarded, and the steroids were then eluted with 5 mL of methanol. The solvents from all fractions were then evaporated under a stream of nitrogen in a water bath at 60°C. The conjugated steroids in Fraction A1 were hydrolyzed by incubation with 10–12 units of *β*-glucuronidase in 1.0 mL of 0.1 M potassium phosphate buffer at 37°C for 18 hr. The now unconjugated androstanediol (adiol) and dehydroepiandrosterone (DHEA) were then extracted from the incubation mixture with ethyl ether (Fraction A2). The ethyl ether was then evaporated under a stream of nitrogen in a water bath at 60°C. 

The estrogens were separated from the neutral steroids in Fraction B1 by solvent partition between 0.4 M aqueous NaOH and toluene. The aqueous fraction was neutralized by the addition of an equivalent amount of HCl, and the estrogens were extracted from the neutralized aqueous solution with ethyl ether (Fraction B2). The toluene was evaporated from the neutral steroids and the ethyl ether was evaporated from the estrogen fraction (Fraction B3). Each of the steroids was later measured by radioimmunoassays with available antibodies and ^3^H-steroids from New England Nuclear Corp, Newton, MA. 

The biologically active fraction of total serum cortisol is free cortisol, but that assay is technically demanding, expensive, and not in general use. Total cortisol is bound to plasma proteins, particularly corticosteroid-binding-globulin (CBG) or sex hormone binding globulin (SHBG), hepatic proteins having estrogen-induced increased synthesis. Intra-assay percentile coefficients of variation (% CV) were all less than 12%, as the criterion for acceptability of measurement results. Too few batches of assays were performed in either of the 1992 or 1994 sets to analyze their interassay variability. The fractionation procedure permitted steroid differentiation as did the antibody specificity incorporated in the assays.

### 2.4. Statistical Methods

Frequency distributions of the hormonal values were examined for acceptability of unimodality and symmetry features. Extreme outliers were observed in several hormones, particularly progesterone, as expected by physiological peaks during the luteal phase, and the opposite near-zero values during the postmenopausal status. Several extreme high outliers were also found in estradiol, which were attributed to ovulatory surges. Extreme outliers were assigned to the upper ranges observed in the population frequency distribution curves, thereby diminishing their statistical influence [26]. Differences in mean values of assayed steroids between the first (1992) and second (1994) sets were normalized, usually adjusting from the smaller first set mean to the larger second set mean, but reversed for Δ4A, corticosterone, deoxycortisol, and DHEAS, in order to eliminate negative normalized values. Natural log conversion was performed on all values to improve their symmetry. Age-adjusted bivariate correlations (Pearson) of the individual steroids were performed on the normalized and log-converted values. These values were further transformed into *z*-scores to reduce their variances (1) in performing *t*-test differences of hormonal levels between subject groups and data subsets, and (2) in scatterplot examinations and confirmations of the data. The *z*-score values of the total normalized subjects centered on 0.00 standard deviations (SDs) and were almost always distributed between ±2 or ±3 SDs. The Fisher *r*-to-*z* transformation was used to estimate significance of differences between two hormonal correlation coefficients in comparisons of total pre-RA versus CN as well as for subjects with lower than grand mean versus higher than grand mean baseline cortisol levels. When a significant difference was found in bivariate correlations between study groups and subgroups, a further correlational analysis was performed using set-specific *z*-scores of the respective reported assays in 1992 and 1994. When both analytical methods revealed significant (*P* ≤ 0.05) differences in correlations, those findings were indicated in the tables. A tentative illustrative model of adrenal glucocorticoid and androgenic anabolic (AA) steroid interrelations was inferred from hormonal differences observed between the total, lower, and higher cortisol subsets. In this exploratory study, a significance level of *P* ≤ 0.050 was accepted without adjustment for multiple comparisons [27].

## 3. Results and Discussion 

### 3.1. Differences in Reported and Normalized Steroids in Pre-RA and CN (Tables 1 and 2)

 The mean values of the normalized hormone assays (combining 1992 and 1994) for the total pre-RA and CN subjects were similar, except for androstenedione (Δ4A), which was lower (*P* = 0.018) in the 28 cases (6.4 ± 0.40) than the 108 CN (7.8 ± 0.28) (Table 1). The first set (1992) pre-RA females contributed mainly to that difference; 5 of those 12 earlier tested cases had Δ4A levels of −1 SD or lower than the total subjects' mean level, compared to 1 of the 16 pre-RA tested in the second set (*P* = 0.057). The lower Δ4A levels were independent of baseline age, RA onset age, or interval in years from cohort entry to RA onset.

 Our recent report [15] indicated that 4 (11.1%) of the 36 pre-RA females versus 1 (0.70%) of the 143 CN had definitely low baseline cortisol levels (<55 nmol/L, *P* = 0.006). Accordingly, further analysis was performed on the study subjects stratified into subgroups of lower (<0.00 SD grand mean *z*-scores) versus higher (0.00 SD or greater) mean cortisol levels of the total 180 females, both subsets having similar mean ages (Table 2). Again, the mean Δ4A level was lower in the pre-RA versus CN, but significant (*P* = 0.013) only in the subset of 9 pre-RA versus 53 CN with lower mean cortisol levels (5.6 ± 0.73 versus 8.0 ± 0.42 nmol/L, resp.). The mean DHEA level was not significantly (*P* = 0.133) lower in 10 pre-RA versus 52 CN having lower mean cortisol levels (14.0 ± 1.06 versus 19.8 ± 1.88, resp.). However, all cases had a negative *z*-score value of <0.00 SD versus 27 (51.9%) of 52 CN (*P* = 0.004). This findings supports a previous report [8] of a greater (*P* = 0.017) proportion of 15 premenopausal RA patients having combined lower DHEAS and cortisol levels than 14 matched control women (40% versus none). The total 12 pre-RA subjects with lower cortisol values in this study had a borderline (*P* = 0.058) lower baseline mean cortisol level than the comparator 72 CN (102.5 ± 14.7 versus 145.0 ± 5.72 nmol/L, resp.).

The mean level of corticosterone (cmpd B), the complementary steroid in the mineralocorticoid pathway to cortisol (Figure 1), was significantly greater in subjects having higher versus lower grand mean cortisol levels, among the CN (*P* = 0.001), pre-RA (*P* = 0.015), and total (*P* < 0.001) females (Table 2). Further, the bivariate correlations of corticosterone and cortisol levels were significant in the total (Table 3) and higher-level cortisol (Table 5) pre-RA and CN subjects, as well as in the lower-level cortisol pre-RA cases (Table 4), implying that these hormone levels may reflect corresponding hypothalamic-pituitary (H-P) activations. Without having the negative feedback control, as exists for cortisol, corticosterone production does respond to interval pulsatile activation of ACTH, and may be an approximate surrogate indicator of variations in H-P stimulation of cortisol levels. The subject group with higher cortisol levels likely represents greater than average adrenal cortical ACTH activation, which is inferred from the conjointly higher levels of cortisol and its non-17-hydroxylated comparable mineralocorticoid (Figure 1).

The mean estradiol (E2) concentration was greater (*P* = 0.007) in the 18 pre-RA subjects who had a higher than grand mean cortisol level than their 50 comparator CN (265 ± 35 versus 222 ± 34 pmol/L, resp.). As a complementary estrogenic finding, the 9 pre-RA who had higher cortisol levels had greater (*P* = 0.033) mean estrone (E1) levels than the 5 cases with lower cortisol (Table 2). Among the CN subjects, however, the mean E2 level was greater (*P* = 0.009) in those who had lower (*n* = 52) versus higher (*n* = 50) baseline mean cortisol levels (283 ± 32 versus 222 ± 34 pmol/L, resp.). 

### 3.2. Bivariate Correlations of Hormones in Total, Lower, and Higher Cortisol Pre-RA versus CN

 The total female pre-RA (top) versus CN (bottom) age-adjusted, mean-normalized, and log-transformed bivariate correlations (Pearson) of the hormone profile are indicated in Table 3. In the CN, as expected in normal physiology (Figure 1), pregnenolone, the initial steroid in the biosynthetic pathway, was strongly (*P* < 0.001) correlated with its proximate products: progesterone, 17-OH pregnenolone, and 17-OH progesterone. However, pregnenolone was not correlated with the adrenal androgens (DHEA and Δ4A), nor with cortisol. Rather, it was correlated with corticosterone (*P* = 0.005) and with deoxycortisol (*P* = 0.004). Of interest, pregnenolone was significantly (*P* = 0.008) correlated with estradiol (E2). In the smaller pre-RA sample, pregnenolone was also correlated significantly with progesterone, 17-OH progesterone, and deoxycortisol, but again, not with cortisol.

 Regarding correlations of the adrenal androgens, DHEA, and Δ4A, each was strongly correlated with the other and with their 17-hydroxylated precursors, 17-OH pregnenolone and 17-OH progesterone, in both the pre-RA and CN subjects (Table 3). These findings indicate that the 17*α*-hydroxylase step is important in AA synthesis (Figure 1). The AAs were not correlated with the nonhydroxylated pregnenolone or progesterone enzymatic step precursors.

### 3.3. Similar Bivariate Correlations in the Total Pre-RA versus CN Subjects (Table 3)

The bivariate hormonal correlations for total pre-RA (top) and CN (bottom) subjects were similar (Table 3). Cortisol and E2 were reported to have counter-opposing effects on their alternate hypothalamic-pituitary (H-P) axis controls [17]. Cortisol and HPA axis stimulation tend to inhibit the HPG axis, whereas estrogen may stimulate the HPA axis and the peripheral production of cortisol [17].

### 3.4. Differences in Bivariate Correlations of Lower Cortisol Pre-RA versus CN Subjects (Table 4)

**Figure 2:**
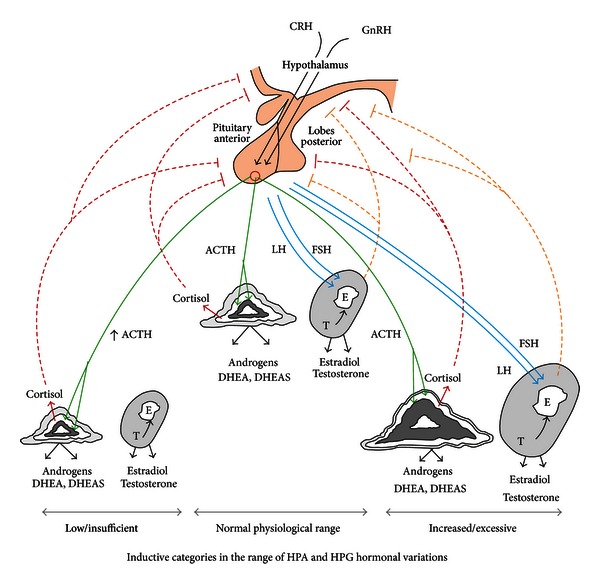
An inductive categorical schema of adrenal cortical and ovarian *tropic* (ACTH, LH, and FSH) and *trophic* (steroidogenic cell mass or competency) influences on cortisol, adrenal androgen (AA), and sex hormone production, in relation to AA status in females. Negative feedback control of the hypothalamic-pituitary-adrenal (HPA) and ovarian (HPG) axes is illustrated. Solid lines indicate stimulation and dashed lines indicate inhibition, within the respective systems. Direct and indirect interactions between the respective HPA and HPG axes are not illustrated. The HPA axis can inhibit the HPG axis at multiple levels and estrogen may stimulate the HPA axis [17]. The enlarged ovary model portrays *tropic* (LH, FSH) and *trophic* mechanisms operating in polycystic ovarian syndrome (PCOS), rather than a normally large gland. The adrenal cortex and ovary are derived from a common embryonic anlage. It is not known if *trophic* influences can cause an “*enlarged adrenal organ syndrome*,” analogous to PCOS. Regarding low adrenal and low ovarian gland sizes, defined syndromes are also not documented, but individual variation and diminishing size occur in natural aging. Cortisol is synthesized mainly in the zona fasciculata (ZF, white area), which has the largest mass of the adrenal cortex (circa 70%). The AAs, DHEA and androstenedione, are mainly produced in the zona reticularis (ZR, darker grey area), which has the smallest mass (circa 10%). In females, the AAs are the major source of androgenic compounds. The mineralocorticoids are synthesized in the outermost zona glomerulosa (ZG, light gray area), which has a medium mass (circa 20%) of the adrenal cortex. Cortisol and AA production are stimulated by ACTH secretion in a pulsatile pattern under regulation of the hypothalamic-pituitary (H-P) axis. Cortisol, in turn, inhibits the H-P in a *negative feedback* manner. It directly suppresses hypothalamic corticotropin releasing hormone (CRH) and its action on the pituitary secretion of ACTH. Insufficiency of cortisol leads to less inhibition of the CRH-ACTH axis and to increased ACTH. In normal aging, cortisol levels are fully maintained in the setting of decreased AA production and associated decreased ZR mass. The overall size of the adrenal cortex is stable in aging, but the outer cortical zones (ZG and ZF) are relatively increased in size to the diminished ZR [18]. The ovarian AA steroids are androstenedione mainly and testosterone (T), which lack negative feedback inhibition at the H-P levels, a function accomplished by estrogen (E).

 In the lower cortisol subject groups (Table 4), expected physiological interrelations were again observed, as described above for the total subjects (Table 3). The bivariate correlations were again generally similar between subject groups, but with 2 differences (deltas). The pre-RA had significantly stronger correlations than the CN in deoxycortisol with 17-OH progesterone (*r* = 0.860, *P* = 0.003, *n* = 10 versus *r* = 0.173, *P* = 0.215, *n* = 54, resp., delta *P* = 0.008) and with Δ4A (*r* = 0.782, *P* = 0.022, *n* = 9 versus *r* = −0.017, *P* = 0.903, *n* = 53, resp., delta *P* = 0.014). Again, these study group correlational differences raise the issue of the degree of ACTH activation of the adrenal cortical pathways, feasibly being stronger in the lower cortisol pre-RA versus CN subjects (Figure 2).

### 3.5. Differences in Bivariate Correlations of Higher Cortisol Pre-RA versus CN Subjects (Table 5)

Concerning the higher cortisol levels (Table 5), almost twice the number of pre-RA subjects were included than in the lower cortisol group (Table 4). Four low-level (*P* ≤ 0.050) significant differences were observed, including 3 pairs of hormonal correlations involving pregnenolone, 17-hydroxypregnenolone, and estrone (E1). In pre-RA, the pregnenolone correlation was negative with cortisol in pre-RA (*r* = −0.469, *P* = 0.037, *n* = 21), but positive in the CN (*r* = 0.186, *P* = 0.169, *n* = 57), the difference being significant (*P* = 0.011). Regarding the hydroxylated precursor steroid, 17-OH pregnenolone, the pre-RA had even stronger positive correlation with DHEA (*r* = 0.842, *P* < 0.000, *n* = 19) than did the CN (*r* = 0.575, *P* < 0.000, *n* = 55), the difference also being significant (*P* = 0.046) (Table 5). The stronger positive AA steroid correlation of DHEA with its precursor steroid, 17-OH pregnenolone, may imply that the observed relative deficiency of this AA had not likely resulted from a specific inhibition at the 17*α*-hydroxylase step (Figure 1). 

The correlations of estrone (E1) with both corticosterone and deoxycortisol were negative in pre-RA (*r* = −0.751 and *r* = −0.676, resp.), but positive in CN (*r* = 0.045 and *r* = 0.217, resp.), the differences being *P* = 0.025 and *P* = 0.023, respectively (Table 5). The mean estrone level was greater (*P* = 0.033) in the pre-RA women with higher versus lower cortisol levels (265 ± 44.3 versus 127 ± 27.3 pmol/L, resp.), as indicated in Table 2. 

### 3.6. Inferences from the Pre-RA versus CN Hormonal Profile Mean Differences and Correlations

 In the absence of quantitative measurements of the degree of ACTH, FSH, and LH end-organ stimulation (Figure 2), interpretation of the baseline serum adrenal and sex steroid profiles is challenging in the pre-RA versus CN subjects of this study as well as in previous comparisons of RA patients and controls [4–8, 12, 28–32]. Adrenal cortical stimulation by the insulin-induced hypoglycemia test (IIHT) was studied in pre-menopausal RA patients and control females. One study [33] did not reveal a significant difference in cortisol response. The other study [8] revealed a baseline combined “lower” quartile range of serum cortisol and DHEAS levels including 6 of the 15 early RA patients, but none of 14 CN (*P* = 0.017). The finding suggested a relative hypocompetence of adrenal cortical function in some premenopausal RA females [8]. Few studies have measured ACTH, serum cortisol, or other steroid levels of RA patients without having glucocorticoid therapy at baseline measurements or after HPA axis stimulation [4, 12, 34–39]. Two studies [34, 36] showed significantly elevated ACTH levels without hypercortisolemia in untreated RA patients, but a difference was not found in the other studies. Lower DHEA levels in two of the above studies suggested decreased synthesis of AAs and deficient zona reticularis function in the RA versus CN [4, 12].

To our knowledge, this is the first study of a comprehensive panel of adrenal and sex steroid levels in women prior to the onset of RA and matched cohort controls. The lower androstenedione level in the pre-RA occurred mainly in those subjects who also had lower cortisol levels than the grand mean of total subjects. In that lower-cortisol subgroup (Table 4), pre-RA had a stronger correlation of deoxycortisol with Δ4A than did the CN. The preceding non-AA steroid level may be a surrogate indicator of ACTH stimulation, which is not directly regulated by the negative feedback mechanism controlling cortisol levels (Figure 2). 

The lower AA levels in a minority of the pre-RA cases may likely reflect a lower AA synthesis capacity in those women, rather than lesser ACTH stimulation. Perhaps, such physiology may be analogous to natural processes in aging [18, 40, 41]? Figure 2 outlines an inductive model in which a minority subset of pre-RA women may have lower or relatively insufficient adrenal cortical functional capacity, rather than a deficit of hypothalamic-pituitary (H-P) stimulation or specific enzymatic defects [41–45]. The mechanisms for biological impairments of AA production are profoundly complex and are not yet fully defined but do occur in natural aging [18, 40, 41]. The biosynthetic mechanisms controlling AA synthesis and their levels during life cycles and between the genders remain unproved [41–45]. Further research is required to confirm the observed relative AA insufficiency in a minority subset of women before onset of RA as well as the differences found in hormonal correlations. If these findings are confirmed, they will need to be defined in terms of their biosynthetic and control mechanisms [18, 40–45].

## 4. Conclusions

 Serum androstenedione (Δ4A) levels were lower in a cohort of pre-RA versus CN females, particularly among subjects who had lower than the total subjects' mean cortisol level (Tables 1 and 2). In pre-RA versus CN women having lower cortisol levels (Table 4), stronger correlations of deoxycortisol with 17-OH progesterone and with Δ4A were found in the cases, suggesting that those subjects had sufficient or potentially increased ACTH stimulation. The pre-RA with higher cortisol levels showed a stronger correlation of DHEA with its 17-OH pregnenolone precursor than did the CN (Table 5). Accordingly, the AA deficiency does not seem likely based upon the respective enzymatic defect. 

Multiple comparisons were performed in this study which could lead to an inflated rate of type I error, identifying low-level (*P* ≤ 0.050) differences which do not truly exist, and which require independent confirmation. Steroid interactions are part of complex homeostatic control mechanisms which tend to modulate deviations. In such systems, accumulated minor deviations in the same direction could be supportive of subtle dysfunctions, as is proposed. However, the observed differences were found only in a minority subgroup of pre-RA women versus CN women. Further investigation is needed of possible polymorphic variations diminishing cellular biosynthetic capacities of adrenal and ovarian steroids as a risk factor for RA in a subset of females (Figure 2).

## Figures and Tables

**Table 1:** Hormones reported by the referral laboratory in a first (1992) and second (1994) set of assays.

Hormones assayed and statistical values	Reported assay results in 1st and 2nd female sets			Normalized combined assays		
	1st Set	2nd Set	All females	Control	Pre-RA	All females
Pregnenolone:						
Mean (nmol/L) ± SE (*n*)	5.3 ± 0.57 (69)	5.2 ± 0.32 (73)*	5.3 ± 0.32 (142)	5.1 ± 0.34 (111)	5.6 ± 0.88 (31)	5.2 ± 0.32 (142)
Median; IQR	3.1; 2.70–5.56	4.6; 3.57–6.95	3.9; 3.11–6.50	3.8; 2.67–6.59	3.95; 3.29–6.06	3.8; 2.93–6.57
Progesterone:						
Mean (nmol/L) ± SE (*n*)	5.8 ± 1.26 (69)	14.0 ± 2.02 (107)	10.8 ± 1.35 (176)	13.4 ± 1.49 (141)	17.3 ± 3.37 (35)	14.2 ± 1.37 (176)
Median; IQR	0.0; 0.00–9.79	3.9; 1.88–19.4	2.9; 0.00–11.5	5.8; 2.10–16.1	5.0; 2.10–30.1	5.7; 2.10–19.3
17-OH Pregnenolone:						
Mean (nmol/L) ± SE (*n*)	5.1 ± 0.40 (69)	7.2 ± 0.57 (73)	6.2 ± 0.36 (142)	7.2 ± 0.41 (111)	7.0 ± 0.71 (31)	7.1 ± 0.35 (142)
Median; IQR	4.4; 2.50–7.34	5.6; 4.21–9.45	5.1; 3.34–7.96	5.9; 4.33–9.27	5.9; 3.82–8.90	6.0; 4.30–9.26
17-OH Progesterone:						
Mean (nmol/L) ± SE (*n*)	3.8 ± 0.36 (69)	4.8 ± 0.53 (73)*	4.3 ± 0.33 (142)	4.8 ± 0.39 (111)	4.5 ± 0.60 (31)	4.7 ± 0.33 (142)
Median; IQR	2.6; 1.66–5.58	3.4; 1.60–6.27	2.8; 1.63–5.85	3.2; 1.93–6.35	3.6; 1.57–6.93	3.5; 1.93–6.38
Dehydroepiandrosterone:						
Mean (nmol/L) ± SE (*n*)	11.5 ± 1.04 (63)	19.8 ± 1.78 (73)	15.9 ± 1.12 (136)	20.2 ± 1.23 (107)	18.0 ± 2.18 (29)	19.7 ± 1.07 (136)
Median; IQR	9.1; 5.28–17.4	15.9; 12.5–19.3	13.4; 8.63–18.4	16.9; 13.4–25.0	15.5; 12.0–17.8	16.3; 13.1–21.8
Androstenedione:						
Mean (nmol/L) ± SE (*n*)	7.5 ± 0.45 (64)	3.2 ± 0.27 (72)	5.2 ± 0.32 (136)	7.8 ± 0.28 (108)	6.4 ± 0.40 (28)^†^	7.5 ± 0.24 (136)
Median; IQR	6.7; 4.90–10.4	2.8; 1.71–4.16	4.2; 2.23–7.25	7.2; 5.91–9.53	6.5; 4.99–7.88	7.1; 5.55–8.97
Testosterone:						
Mean (nmol/L) ± SE (*n*)	0.89 ± 0.05 (60)	2.5 ± 0.21 (73)	1.8 ± 0.13 (133)	2.5 ± 0.14 (103)	2.5 ± 0.21 (30)	2.5 ± 0.12 (133)
Median; IQR	0.83; 0.56–1.11	2.3; 1.39–3.35	1.3; 0.76–2.34	2.4; 1.80–3.03	2.3; 1.84–2.89	2.4; 1.81–2.95
Estradiol:						
Mean (pmol/L) ± SE (*n*)	562 ± 29 (69)	263 ± 42.3 (61)	422 ± 28 (130)	253 ± 24 (102)	247 ± 43 (28)	252 ± 21 (130)
Median; IQR	518; 435–659	159; 75–315	386; 175–568	202; 96–329	181; 108–367	191; 98–340
Estrone:						
Mean (pmol/L) ± SE (*n*)	282 ± 43 (69)	Not performed	283 ± 43 (69)	289 ± 53 (55)	216 ± 35 (14)	274 ± 43 (69)
Median; IQR	209; 146–309		209; 146–309	201; 140–301	182; 122–297	201; 137.0–300
Corticosterone (cmpd B):						
Mean (nmol/L) ± SE (*n*)	10.2 ± 0.85 (69)	1.63 ± 0.17 (110)	5.0 ± 0.47 (179)	10.5 ± 0.40 (143)	9.6 ± 0.64 (36)	10.3 ± 0.34 (179)
Median; IQR	8.00; 6.23–12.8	1.18; 0.45–2.00	2.28; 0.92–7.36	9.60; 8.56–11.0	9.54; 7.76–10.7	9.60; 8.56–10.9
Deoxycortisol:						
Mean (nmol/L) ± SE (*n*)	2.3 ± 0.18 (69)	0.49 ± 0.09 (110)	1.2 ± 0.11 (179)	2.3 ± 0.10 (143)	2.2 ± 0.17 (36)	2.3 ± 0.09 (179)
Median; IQR	2.2; 1.07–3.15	0.25; 0.00–0.67	0.64; 0.12–1.85	2.2; 1.6–2.7	2.1; 1.57–2.93	2.2; 1.6–2.7
Cortisol:						
Mean (nmol/L) ± SE (*n*)	280.0 ± 17 (69)	236.4 ± 12 (110)	253.1 ± 10 (179)	233.7 ± 11 (143)	245.2 ± 24 (36)	236.0 ± 9.9 (179)
Median; IQR	250.8; 189–327	218.1; 148–286	232.0; 164–309	206.1; 155–285	240.5; 143–329	217.4; 154–286
DHEA sulfate (DHEAS):						
Mean (*μ*mol/L) ± SE (*n*)	2.9 ± 0.27; (69)	1.9 ± 0.14 (110)	2.4 ± 0.14 (179)	2.9 ± 0.16 (143)	2.5 ± 0.26 (36)	2.9 ± 0.14 (179)
Median; IQR	2.4; 1.24–4.20	1.8; 1.04–2.63	1.9; 1.10–3.08	2.6; 1.63–3.86	2.3; 1.53–3.50	2.5; 1.59–3.83
Mean ages ± SEs	**43.7 ± 1.2**	**44.0 ± 1.2**	**43.9 ± 0.89**	**43.9 ± 0.99**	**43.8 ± 2.03**	**43.9 ± 0.89**

SE: standard error of mean; IQR: interquartile range of median.

*Mean steroid values which did not differ (*P* > 0.050) between sets (see text for details). ^†^ *P* = 0.018.

**Table 2:** Normalized assays in CN and Pre-RA Subjects having lower versus higher cortisol values than the total female mean.

Hormones assayed and statistical values	Normalized assays in low cortisol females			Normalized assays in high cortisol females		
	Control	Pre-RA	Total	Control	Pre-RA	Total
Pregnenolone:						
Mean (nmol/L) ± SE (*n*)	5.1 ± 0.49 (54)	4.9 ± 1.08 (10)	5.1 ± 0.44 (64)	5.1 ± 0.47 (57)	5.9 ± 1.20 (21)	5.3 ± 0.47 (78)
Median; IQR	4.0; 2.88–6.05	3.8; 2.87–6.18	3.9; 2.96–5.79	3.6; 2.61–7.08	4.2; 3.47–6.49	3.8; 2.87–6.94
Progesterone:						
Mean (nmol/L) ± SE (*n*)	12.4 ± 1.9 (71)	18.3 ± 7.10 (12)	13.2 ± 1.98 (83)	14.5 ± 2.22 (70)	16.8 ± 3.68 (23)	15.0 ± 1.90 (93)
Median; IQR	5.5 ± 2.10–13.6	4.7; 2.10–36.2	4.8; 2.10–16.2	5.9; 2.15–17.0	12.7; 2.77–30.1	6.1; 2.29–22.5
17-OH Pregnenolone:						
Mean (nmol/L) ± SE (*n*)	6.9 ± 0.60 (54)	5.7 ± 0.74 (10)	6.7 ± 0.52 (64)	7.5 ± 0.55 (57)	7.5 ± 0.98 (21)	7.5 ± 0.48 (78)
Median; IQR	5.7; 4.49–8.74	6.1; 2.97–7.89	5.8; 4.33–8.12	6.7; 4.26–9.84	5.7; 4.17–9.58	6.0; 4.27–9.78
17-OH Progesterone:						
Mean (nmol/L) ± SE (*n*)	4.7 ± 0.49 (54)	4.9 ± 1.12 (10)	4.7 ± 0.45 (64)	4.9 ± 0.61 (57)	4.3 ± 0.72 (21)	4.8 ± 0.48 (78)
Median; IQR	3.22; 1.98–6.50	4.34; 1.55–7.77	3.51; 1.95–7.00	3.23; 1.78–6.16	3.51; 1.70–6.11	3.45; 1.86–6.10
Dehydroepiandrosterone:						
Mean (nmol/L) ± SE (*n*)	19.8 ± 1.88 (52)	14.0 ± 1.06 (10)	18.9 ± 1.60 (62)	20.5 ± 1.62 (55)	20.1 ± 3.21 (19)	20.4 ± 1.45 (74)
Median; IQR	16.9; 13.5–21.8	14.2; 11.9–17.4	16.5; 12.5–19.9	16.2; 13.4–26.1	16.1; 11.5–20.9	16.1; 13.2–26.0
Androstenedione:						
Mean (nmol/L) ± SE (*n*)	8.0 ± 0.42 (53)	5.6 ± 0.73 (9)*	7.7 ± 0.39 (62)	7.6 ± 0.38 (55)	6.7 ± 0.47 (19)	7.4 ± 0.31 (74)
Median; IQR	7.2; 6.20–9.69	6.6; 3.85–6.87	7.1; 6.12–9.00	7.2; 5.63–9.54	6.4; 5.18–8.65	7.1; 5.42–8.88
Testosterone:						
Mean (nmol/L) ± SE (*n*)	2.7 ± 0.22 (51)	2.5 ± 0.33 (10)	2.7 ± 0.19 (61)	2.3 ± 0.16 (52)	2.4 ± 0.28 (20)	2.3 ± 0.14 (72)
Median; IQR	2.4; 2.02–3.13	2.3; 1.79–3.09	2.4; 2.02–3.04	2.3; 1.67–2.99	2.2; 1.82–2.90	2.3; 1.73–2.91
Estradiol:						
Mean (pmol/L) ± SE (*n*)	283 ± 32 (52)†	215 ± 105 (10)	272 ± 31 (62)	222 ± 34 (50)	265 ± 35 (18)^†^	233 ± 27 (68)
Median; IQR	221; 133–335	128; 3.9–251	214; 113–296	139; 56–328	195; 152–414	175; 62–354
Estrone:						
Mean (pmol/L) ± SE (*n*)	330 ± 102 (28)	127 ± 27.3 (5)	299 ± 87.2 (33)	246 ± 26.9 (27)	265 ± 44.3 (9)*	251 ± 22.7 (36)
Median; IQR	199; 135–293	141; 65–182	195; 120–274	208; 141–330	269; 134–366	218.; 141–353
Corticosterone (cmpd B):						
Mean (nmol/L) ± SE (*n*)	9.3 ± 0.3 (72)	7.3 ± 0.9 (12)	9.1 ± 0.3 (84)	11.7 ± 0.7 (71)^‡^	10.7 ± 0.8 (24)*	11.4 ± 0.6 (95)^‡^
Median; IQR	9.2; 8.35–10.3	9.0; 4.43–9.64	9.2; 8.19–10.1	10.2; 9.01–12.8	10.1; 8.91–11.6	10.2; 8.95–12.4
Deoxycortisol:						
Mean (nmol/L) ± SE (*n*)	2.3 ± 0.13 (72)	2.1 ± 0.29 (12)	2.3 ± 0.12 (84)	2.2 ± 0.16 (71)	2.2 ± 0.22 (24)	2.2 ± 0.13 (95)
Median; IQR	2.3; 1.69–2.63	2.0; 1.57–3.02	2.3; 1.65–2.63	2.0; 1.55–2.66	2.2; 1.57–2.93	2.0; 1.55–2.66
Cortisol:						
Mean (nmol/L) ± SE (*n*)	145.0 ± 5.7 (72)	102.5 ± 14.7 (12)	138.9 ± 5.5 (84)	323.7 ± 14.9 (71)	316.5 ± 23.7 (24)	321.9 ± 12.6 (95)
Median; IQR	156; 111–179	113.6; 51–146	148.0; 105–174	285.3; 241–351	280.3; 237–375	285.3; 241–361
DHEA sulfate (DHEAS):						
Mean (*μ*mol/L) ± SE (*n*)	3.0 ± 0.24 (72)	2.1 ± 0.50 (12)	2.9 ± 0.22 (84)	2.9 ± 0.21 (71)	2.6 ± 0.30 (24)	2.8 ± 0.17 (95)
Median; IQR	2.53; 1.70–3.73	1.8; 0.79–4.02	2.5; 1.58–3.73	2.6; 1.59–3.99	2.3; 1.60–3.49	2.4; 1.59–3.91
Mean ages ± SEs^‡^	**43.3 ± 1.4**	**44.6 ± 3.4**	**43.5 ± 1.3**	**44.6 ± 1.4**	**43.4 ± 2.5**	**44.3 ± 1.2**

SE: standard error of mean; IQR: interquartile range of median.

*P ≤ 0.050; ^†^ *P* ≤ 0.010; ^‡^ *P* ≤ 0.001 (see text for noncortisol differences between low versus high females and in pre-RA versus CN).

**Table 3:** Age-adjusted, log-normalized correlations (Pearson) of total female pre-RA (top) and CN (bottom) hormone levels*.

Hormones		Preg	Prog	17-OHpreg	17-OHprog	DHEA	Adione	Test	Estradiol	Estrone	Cmpd B	DeoxyCort	Cortisol	DHEAS
Preg	*r*		0.532	0.149	0.476	0.025	0.163	−0.195	0.305	−0.081	0.097	0.430	−0.011	0.085
	*p*		0.003	0.432	0.008	0.898	0.417	0.310	0.122	0.794	0.611	0.018	0.954	0.657
	*n*		30	31	31	29	28	30	28	14	31	31	31	31

Prog	*r*	0.516		0.301	0.681	0.071	0.162	−0.512	0.266	0.021	0.122	0.349	−0.200	0.248
	*p*	0.000		0.113	0.000	0.726	0.428	0.005	0.189	0.947	0.491	0.043	0.258	0.158
	*n*	111		30	30	28	27	29	27	14	35	35	35	35

17-OHpreg	*r*	0.392	0.315		0.629	0.785	0.666	0.061	0.264	0.273	0.203	0.194	0.074	0.419
	*p*	0.000	0.001		0.000	0.000	0.000	0.753	0.184	0.366	0.281	0.305	0.696	0.021
	*n*	111	111		31	29	28	30	28	14	31	31	31	31

17-OHprog	*r*	0.529	0.681	0.724		0.344	0.452	−0.381	0.367	−0.071	0.046	0.400	−0.237	0.274
	*p*	0.000	0.000	0.000		0.074	0.018	0.041	0.060	0.817	0.809	0.028	0.207	0.142
	*n*	111	111	111		29	28	30	28	14	31	31	31	31

DHEA	*r*	0.126	0.021	0.624	0.419		0.643	0.173	0.136	0.251	0.274	0.228	0.184	0.489
	*p*	0.199	0.828	0.000	0.000		0.000	0.377	0.518	0.457	0.158	0.244	0.350	0.008
	*n*	107	107	107	107		28	29	26	12	29	29	29	29

Adione	*r*	0.163	0.075	0.427	0.357	0.762		0.014	0.542	0.216	0.506	0.328	0.174	0.625
	*p*	0.093	0.443	0.000	0.000	0.000		0.944	0.006	0.524	0.007	0.094	0.384	0.000
	*n*	108	108	108	108	107		28	25	12	28	28	28	28

Test	*r*	−0.01	−0.254	0.256	−0.047	0.411	0.306		−0.18	−0.123	0.036	0.042	0.049	0.009
	*p*	0.922	0.010	0.009	0.638	0.000	0.002		0.380	0.704	0.852	0.830	0.800	0.962
	*n*	103	103	103	103	102	103		27	13	30	30	30	30

Estradiol	*r*	0.264	0.281	0.196	0.363	0.112	0.204	0.00		0.485	0.485	0.213	0.462	0.120
	*p*	0.008	0.004	0.049	0.000	0.273	0.044	0.999		0.093	0.011	0.285	0.015	0.550
	*n*	102	102	102	102	98	99	94		14	28	28	28	28

Estrone	*r*	0.127	0.077	−0.191	−0.136	−0.044	−0.01	−0.011	0.323		0.033	−0.403	0.387	−0.087
	*p*	0.361	0.579	0.167	0.327	0.761	0.943	0.941	0.017		0.914	0.172	0.191	0.778
	*n*	55	55	55	55	51	52	47	55		14	14	14	14

Cmpd B	*r*	0.267	0.133	0.176	0.078	−0.076	0.027	0.048	0.025	−0.074		0.510	0.579	0.21
	*p*	0.005	0.117	0.065	0.416	0.441	0.786	0.629	0.802	0.594		0.002	0.000	0.227
	*n*	111	141	111	111	107	108	103	102	55		36	36	36

DeoxyCort	*r*	0.270	0.195	0.100	0.119	−0.013	0.010	0.024	0.288	0.007	0.517		−0.064	0.178
	*p*	0.004	0.021	0.301	0.214	0.895	0.921	0.808	0.003	0.959	0.000		0.713	0.306
	*n*	111	141	111	111	107	108	103	102	55	143		36	36

Cortisol	*r*	−0.026	−0.126	0.131	−0.121	0.089	−0.001	0.028	−0.273	0.047	0.295	−0.053		−0.081
	*p*	0.785	0.139	0.173	0.206	0.366	0.991	0.777	0.006	0.738	0.000	0.528		0.644
	*n*	111	141	111	111	107	108	103	102	55	143	143		36

DHEAS	*r*	0.228	0.074	0.334	0.217	0.460	0.404	0.089	0.087	0.088	0.034	0.039	−0.061	
	*p*	0.017	0.388	0.000	0.022	0.000	0.000	0.373	0.385	0.525	0.687	0.642	0.473	
	*n*	111	141	111	111	107	108	103	102	55	143	143	143	

*No significant difference found between pre-RA versus CN correlations, when also requiring set-specific Z-score values for combining assay results in study subjects.

**Table 4:** Age-adjusted, log-normalized correlations (Pearson) of hormones in lower-level cortisol pre-RA (top) and CN (bottom) females*.

Hormones		Preg	Prog	17-OHpreg	17-OHprog	DHEA	Adione	Test	Estradiol	Estrone	Cmpd B	DeoxyCort	Cortisol	DHEAS
Preg	*r*		0.456	0.179	0.498	0.505	0.442	−0.099	0.489	−0.797	0.418	0.639	0.256	0.662
	*p*		0.217	0.645	0.173	0.165	0.273	0.800	0.182	0.203	0.263	0.064	0.507	0.052
	*n*		10	10	10	10	9	10	10	5	10	10	10	10

Prog	*r*	0.654		0.354	0.712	0.354	0.268	−0.447	0.084	−0.080	0.098	0.658	−0.332	0.665
	*p*	0.000		0.350	0.031	0.350	0.521	0.228	0.831	0.920	0.775	0.028	0.319	0.026
	*n*	54		10	10	10	9	10	10	5	12	12	12	12

17-OHpreg	*r*	0.332	0.285		0.690	0.369	0.571	−0.349	0.336	0.498	−0.019	0.469	−0.130	0.163
	*p*	0.015	0.039		0.040	0.328	0.139	0.358	0.376	0.502	0.962	0.202	0.738	0.676
	*n*	54	54		10	10	9	10	10	5	10	10	10	10

17-OHprog	*r*	0.593	0.729	0.727		0.506	0.656	−0.347	0.536	0.105	0.142	** 0.860** ^†^	−0.143	0.393
	*p*	0.000	0.000	0.000		0.164	0.077	0.360	0.136	0.895	0.716	**0.003**	0.713	0.295
	*n*	54	54	54		10	9	10	10	5	10	**10**	10	10

DHEA	*r*	0.075	0.014	0.664	0.369		0.534	0.512	0.306	−0.029	0.300	0.734	0.077	0.555
	*p*	0.600	0.922	0.000	0.008		0.173	0.159	0.423	0.971	0.432	0.024	0.844	0.121
	*n*	52	52	52	52		9	10	10	5	10	10	10	10

Adione	*r*	0.073	0.077	0.462	0.376	0.796		0.286	0.825	−0.042	0.806	0.782*	0.159	0.212
	*p*	0.606	0.589	0.001	0.006	0.000		0.493	0.012	0.958	0.016	**0.022**	0.707	0.614
	*n*	50	53	53	53	52		9	9	5	9	**9**	9	9

Test	*r*	0.067	−0.234	0.282	0.037	0.447	0.377		−0.037	−0.153	0.198	0.021	0.096	−0.113
	*p*	0.643	0.103	0.047	0.801	0.001	0.007		0.925	0.847	0.609	0.958	0.805	0.773
	*n*	48	51	51	51	50	51		10	5	10	10	10	10

Estradiol	*r*	0.275	0.373	0.287	0.421	0.024	0.012	−0.039		−0.033	0.643	0.553	0.357	0.023
	*p*	0.051	0.007	0.041	0.002	0.871	0.935	0.791		0.967	0.062	0.122	0.345	0.954
	*n*	49	52	52	52	50	51	49		5	10	10	10	10

Estrone	*r*	0.124	−0.030	−0.201	−0.227	−0.194	−0.066	−0.064	0.337		−0.562	−0.155	−0.212	−0.455
	*p*	0.539	0.883	0.315	0.256	0.353	0.748	0.765	0.086		0.438	0.845	0.788	0.545
	*n*	25	28	28	28	26	27	25	28		5	5	5	5

Cmpd B	*r*	0.256	0.036	0.155	0.150	−0.026	0.041	0.112	0.013	−0.248		0.397	0.604	0.231
	*p*	0.064	0.768	0.268	0.283	0.858	0.773	0.441	0.926	0.212		0.227	0.049	0.493
	*n*	54	71	54	54	52	53	51	52	28		12	12	12

DeoxyCort	*r*	0.340	0.207	0.207	**0.173**	0.077	**−0.017**	0.168	0.082	−0.254	0.408		−0.031	0.561
	*p*	0.013	0.086	0.137	**0.215**	0.590	**0.903**	0.242	0.568	0.202	0.000		0.929	0.073
	*n*	54	71	54	**54**	52	**53**	51	52	28	72		12	12

Cortisol	*r*	−0.267	−0.313	0.087	−0.151	0.180	0.127	0.078	−0.205	0.222	−0.012	−0.136		−0.144
	*p*	0.053	0.008	0.534	0.280	0.207	0.369	0.590	0.149	0.265	0.922	0.259		0.673
	*n*	54	71	54	54	52	53	51	52	28	72	72		12

DHEAS	*r*	0.278	0.192	0.535	0.297	0.436	0.299	0.144	0.079	−0.077	0.107	0.082	−0.065	
	*p*	0.044	0.111	0.000	0.031	0.001	0.031	0.318	0.579	0.703	0.372	0.495	0.593	
	*n*	54	71	54	54	52	53	51	52	28	72	72	72	

*Significant differences between pre-RA versus CN correlations, when requiring both adjusted and set-specific Z-score values to combine assay results.

Symbols placed at the greater *r* values: *P ≤ 0.050; ^†^ *P* ≤ 0.010.

**Table 5:** Age-Adjusted, log-normalized correlations (Pearson) of hormones in higher-level cortisol pre-RA (top) and CN (bottom) females*.

Hormones		Preg	Prog	17-OHpreg	17-OHprog	DHEA	Adione	Test	Estradiol	Estrone	Cmpd B	DeoxyCort	Cortisol	DHEAS
Preg	*r*		0.568	0.085	0.485	−0.125	−0.209	−0.201	0.040	0.016	−0.272	0.286	−0.469^†^	−0.310
	*p*		0.011	0.722	0.030	0.621	0.404	0.408	0.880	0.970	0.246	0.222	0.037	0.183
	*n*		20	21	21	19	19	20	18	9	21	21	21	21

Prog	*r*	0.361		0.265	0.689	−0.016	0.021	−0.532	0.499	−0.097	0.100	0.228	−0.485	0.014
	*p*	0.006		0.273	0.001	0.950	0.936	0.023	0.049	0.820	0.659	0.308	0.022	0.952
	*n*	57		20	20	18	18	19	17	9	23	23	23	23

17-OHpreg	*r*	0.441	0.324		0.660	0.842*	0.775	0.182	−0.032	0.279	0.183	0.095	−0.084	0.468
	*p*	0.001	0.015		0.002	**0.000**	0.000	0.456	0.904	0.504	0.439	0.691	0.725	0.037
	*n*	57	57		21	**19**	19	20	18	9	21	21	21	21

17-OHprog	*r*	0.456	0.625	0.728		0.409	0.443	−0.408	0.114	−0.004	0.041	0.234	−0.516	0.232
	*p*	0.000	0.000	0.000		0.092	0.066	0.083	0.662	0.992	0.864	0.320	0.020	0.324
	*n*	57	57	57		19	19	20	18	9	21	21	21	21

DHEA	*r*	0.173	0.003	**0.575**	0.464		0.734	0.192	−0.263	0.333	0.167	0.129	−0.046	0.535
	*p*	0.210	0.981	**0.000**	0.000		0.001	0.446	0.343	0.519	0.508	0.611	0.857	0.022
	*n*	55	55	**55**	55		19	19	16	7	19	19	19	19

Adione	*r*	0.252	0.070	0.405	0.333	0.736		−0.006	−0.020	0.306	0.106	−0.099	−0.098	0.741
	*p*	0.066	0.617	0.002	0.014	0.000		0.980	0.943	0.556	0.677	0.696	0.699	0.000
	*n*	55	55	55	55	55		19	16	7	19	19	19	19

Test	*r*	−0.078	−0.260	0.265	−0.112	0.393	0.232		−0.386	−0.216	0.093	0.103	0.292	0.113
	*p*	0.586	0.065	0.060	0.434	0.004	0.101		0.140	0.642	0.705	0.676	0.225	0.647
	*n*	52	52	52	52	52	52		17	8	20	20	20	20

Estradiol	*r*	0.281	0.301	0.193	0.320	0.226	0.331	−0.043		0.739	−0.228	−0.109	−0.144	0.018
	*p*	0.051	0.036	0.184	0.025	0.126	0.023	0.780		0.036	0.378	0.678	0.581	0.947
	*n*	50	50	50	50	48	48	45		9	18	18	18	18

Estrone	*r*	0.125	0.250	−0.196	0.040	0.146	0.060	0.064	0.402		−0.751*	−0.676*	−0.075	−0.061
	*p*	0.543	0.218	0.338	0.848	0.497	0.782	0.782	0.042		0.032	0.066	0.861	0.886
	*n*	27	27	27	27	25	25	22	27		9	9	9	9

Cmpd B	*r*	0.290	0.165	0.147	0.015	−0.158	0.038	0.088	0.117	0.045		0.612	0.382	0.020
	*p*	0.030	0.175	0.280	0.911	0.253	0.787	0.541	0.424	0.828		0.002	0.072	0.928
	*n*	57	70	57	57	55	55	52	50	27		24	24	24

DeoxyCort	*r*	0.211	0.186	0.023	0.051	−0.087	0.009	−0.082	0.341	0.217	0.631		−0.109	−0.101
	*p*	0.118	0.125	0.865	0.711	0.531	0.947	0.566	0.016	0.286	0.000		0.620	0.646
	*n*	57	70	57	57	55	55	52	50	27	71		24	24

Cortisol	*r*	0.186	−0.244	0.058	−0.274	−0.072	−0.036	0.307	−0.125	−0.252	0.292	0.148		−0.249
	*p*	0.169	0.043	0.669	0.041	0.606	0.798	0.028	0.394	0.214	0.014	0.222		0.252
	*n*	57	70	57	57	55	55	52	50	27	71	71		24

DHEAS	*r*	0.174	−0.078	0.124	0.139	0.482	0.517	0.025	0.139	0.386	−0.035	0.006	−0.222	
	*p*	0.201	0.525	0.362	0.309	0.000	0.000	0.863	0.341	0.052	0.772	0.962	0.065	
	*n*	57	70	57	57	55	55	52	50	27	71	71	71	

*Significant differences between pre-RA versus CN correlations, when requiring both adjusted and set-specific Z-score values to combine assay results.

Symbols placed at the greater *r* values: *P ≤ 0.050; ^†^ *P* ≤ 0.010.
